# A War Office Advisory Committee for the Voluntary Aid Detachments

**Published:** 1910-08-06

**Authors:** 


### The Royal Army Medical Corps Section.

### A WAR OFFICE ADVISORY COMMITTEE FOR THE VOLUNTARY AID DETACHMENTS.

In our issue of July 23 we drew attention to the subject of the Voluntary Aid detachments which are in the course of formation throughout the United Kingdom for completing the Medical Service of the Territorial Army, and we expressed the view that the British Red Cross Society, in agreeing to form these detachments, had perhaps undertaken more than it was in a position to carry out. What seemed to us one of the main difficulties in the successful accomplishment of the scheme was the heavy pecuniary drain that it entailed on the funds of the Red Cross Society, necessitating a special appeal for support in the way of increased membership. Undoubtedly the money question is a very important element in organising this special form of voluntary assistance to our Territorial Army, and Government grants should have been provided for it; but it would seem that other difficulties have presented themselves, and they are of such a nature that they materially affect the success of the movement. So impressed are the military authorities by the reality of these hindrances that they have formed at the War Office an Advisory Committee to facilitate the working of the scheme for the organisation in England and Wales of voluntary aid to the sick and wounded in time of invasion. The committee is composed of three representatives from the War Office, two from the British Red Cross Society, two from the Council of County Territorial Associations, one from the Scottish Branch of the British Red Cross Society, and one from the St. Andrew's Ambulance Association. The St. John Ambulance Association was invited to send two representatives, but did not accept the invitation.

### The Present Position.

To understand the present position of matters it is necessary to recall the fact that, as the outcome of negotiations in 1908 between the War Office and the British Red Cross Society on the subject of the organisation of voluntary aid for the sick and wounded in the event of war in the United Kingdom, the British Red Cross Society not only offered to place at the disposal of the War Office the machinery of its county and other committees for this work, but also undertook to develop its county organisation on a large scale, so as to comprehend every district in the country. This patriotic offer was accepted by the War Office, and in 1908 the Army Council sent out a circular memorandum, No. 31, to the various County Associations, urging them to come into touch with the county and other committees of the British Red Cross Society with the object of co-operation in this important work of rendering assistance to our sick and wounded Territorial soldiers in case of invasion. By the issue of this circular memorandum there was undoubtedly given to the British Red Cross Society by the War Office an official status in connection with this important subject of voluntary aid to the Territorial Army in the event of war in our home territory that marked it out as the body selected for the work. Having settled this point, the next duty of the War Office was to decide what was the best plan on which to organise this voluntary aid. After full consideration the scheme adopted was that of Voluntary Aid detachments, and on August 16, 1909, the Secretary of State for War issued to secretaries of Territorial County Associations in England and Wales a memorandum embodying the details of it, following it up in December 1909 by a separate one for Scotland based on the same lines but differing in certain particulars. It is with the issue of this memorandum that difficulties arose, and the success of the scheme has been to some extent threatened. Upon this a word of explanation is necessary.

### What are Voluntary Aid Detachments?

Briefly stated, the work of the Voluntary Aid detachments is the removal of the sick and wounded from the field ambulances to the general hospitals, and it is necessary that their personnel be trained for the discharge of these duties. The War Office Memorandum for England and Wales lays it down that, members wishing to enrol in the detachments must have in the case of the men a First Aid certificate of the St. John Ambulance Association, and in the case of the women a First Aid and Home Nursing Certificate from the same body. Without these qualifications they are not eligible to join the detachments. In insisting on this preliminary qualification before being eligible for enrolment, the memorandum seriously, in our opinion, handicapped the success of the movement. In the first place, to insist on all those desirous of joining the Voluntary Aid detachments being efficient in First Aid and nursing before enrolment was an unwise and discouraging demand, for it only led to reluctance to join on the part of willing workers who had not the necessary certificates. Naturally they hesitated to undertake all the labour of attending courses of lectures and the anxiety of the subsequent examinations, with the possibility before them of failing to satisfy the examiners. No Territorial regiment would be raised were the possession of a musketry certificate made a preliminary qualification for admission. Again, why should cooks, clerks, carpenters or mechanics be compelled to qualify in courses of instruction that would be of no help to them in their work? Why, too, should members who had First-Aid certificates from reliable teaching bodies, such as the London County Council, the National Health Society and other similar bodies, be ineligible for enrolment, and be compelled to take again a course of First-Aid instruction under the St. John Ambulance Association? By recognising such certificates the standard of First-Aid knowledge of the personnel of the detachments would not be lowered, as they are quite on a level with those issued by the St. John Association. These were some of the difficulties the British Red Cross Society had to face when first forming the detachments, and for them it was in no way responsible. At first there was a reluctance on the part of the War Office to modify the conditions of qualification for the detachments, possibly from pressure put on it by the St. John Ambulance Association; but in a public and patriotic movement such as this there should be no monopoly in the qualifying certificates, and every teaching body should be encouraged in its work of First-Aid instruction. No doubt a teaching body in First Aid, as the St. John's Ambulance Association, was entitled to first consideration in the matter of instructing the personnel of the Voluntary Aid detachments, and the British Red Cross Society did right in securing its help in the preliminary training of candidates wishing to take part in this voluntary aid work, but nothing would justify a monopoly being given to the St. John's and the exclusion of other bodies that give First-Aid certificates.

By the appointing of this Advisory Committee for the Voluntary Aid detachments the War Office has taken a very wise step. It is not to have any executive authority, and its function will be to advise on matters of general policy as regards the working of the scheme. Seeing that it has on it representatives from all the bodies interested in the matter, its deliberations should be of great assistance, and already we understand important modifications have been made in the original memorandum as the result of two meetings that have been recently held. We think it unfortunate that the St. John Ambulance Association did not accept the invitation given to send two representatives to the Advisory Committee, nor can we approve of the recent action of the Association in organising St. John Ambulance County Companies for aid to the sick and wounded of the Territorial Force in the event of war. By doing so it is putting itself into competition with the British Red Cross Society in a matter specially handed over to the latter by the War Office, and it is thus introducing an element of confusion into a scheme which is of vital importance to the Territorial Force and to the welfare of the nation. We understand that a recent communication from the War Office lays it down that no official cognizance is to be given by the military authorities to any body or association which aims at carrying out the work for which the recognised Voluntary Aid detachments are organised. In view of this confirmation of its previous mandate to the British Red Cross Society the St. John Ambulance Association should reconsider its recent line of action and not put itself into opposition to a kindred body. In face of considerable difficulties the British Red Cross Society has already formed 130 Voluntary Aid detachments in the United Kingdom, with a membership of 4,005, and it should be allowed a fair field for carrying out the duty entrusted to it. What has been already accomplished indicates that it can do so.

